# Multiple Genome Segments Determine Virulence of Bluetongue Virus Serotype 8

**DOI:** 10.1128/JVI.00395-15

**Published:** 2015-03-11

**Authors:** Anna Janowicz, Marco Caporale, Andrew Shaw, Salvatore Gulletta, Luigina Di Gialleonardo, Maxime Ratinier, Massimo Palmarini

**Affiliations:** a MRC-University of Glasgow Centre for Virus Research, Glasgow, United Kingdom; b Istituto Zooprofilattico Sperimentale dell'Abruzzo e del Molise G. Caporale, Teramo, Italy

## Abstract

Bluetongue virus (BTV) causes bluetongue, a major hemorrhagic disease of ruminants. In order to investigate the molecular determinants of BTV virulence, we used a BTV8 strain minimally passaged in tissue culture (termed BTV8_L_ in this study) and a derivative strain passaged extensively in tissue culture (BTV8_H_) in *in vitro* and *in vivo* studies. BTV8_L_ was pathogenic in both IFNAR^−/−^ mice and in sheep, while BTV8_H_ was attenuated in both species. To identify genetic changes which led to BTV8_H_ attenuation, we generated 34 reassortants between BTV8_L_ and BTV8_H_. We found that partial attenuation of BTV8_L_ in IFNAR^−/−^ mice was achieved by simply replacing genomic segment 2 (Seg2, encoding VP2) or Seg10 (encoding NS3) with the BTV8_H_ homologous segments. Fully attenuated viruses required at least two genome segments from BTV8_H_, including Seg2 with either Seg1 (encoding VP1), Seg6 (encoding VP6 and NS4), or Seg10 (encoding NS3). Conversely, full reversion of virulence of BTV8_H_ required at least five genomic segments of BTV8_L_. We also demonstrated that BTV8_H_ acquired an increased affinity for glycosaminoglycan receptors during passaging in cell culture due to mutations in its VP2 protein. Replication of BTV8_H_ was relatively poor in interferon (IFN)-competent primary ovine endothelial cells compared to replication of BTV8_L_, and this phenotype was determined by several viral genomic segments, including Seg4 and Seg9. This study demonstrated that multiple viral proteins contribute to BTV8 virulence. VP2 and NS3 are primary determinants of BTV pathogenesis, but VP1, VP5, VP4, VP6, and VP7 also contribute to virulence.

**IMPORTANCE** Bluetongue is one of the major infectious diseases of ruminants, and it is listed as a notifiable disease by the World Organization for Animal Health (OIE). The clinical outcome of BTV infection varies considerably and depends on environmental and host- and virus-specific factors. Over the years, BTV serotypes/strains with various degrees of virulence (including nonpathogenic strains) have been described in different geographical locations. However, no data are available to correlate the BTV genotype to virulence. This study shows that BTV virulence is determined by different viral genomic segments. The data obtained will help to characterize thoroughly the pathogenesis of bluetongue. The possibility to determine the pathogenicity of virus isolates on the basis of their genome sequences will help in the design of control strategies that fit the risk posed by new emerging BTV strains.

## INTRODUCTION

Bluetongue is one of the major infectious diseases of ruminants and is caused by bluetongue virus (BTV), an arbovirus transmitted by Culicoides spp. (1–3). BTV is a member of the Orbivirus genus within the Reoviridae. The double-stranded RNA (dsRNA) genome of BTV is composed of 10 segments encoding seven structural (VP1 to VP7) and four nonstructural (NS1 to NS4) proteins (4, 5). The BTV virion is an icosahedral particle assembled in a triple layer of capsid shells (6, 7). The viral core contains linear dsRNA genomic segments associated with replication complexes formed by three minor proteins, VP1 (RNA-dependent RNA polymerase), VP4 (capping enzyme including methyltransferase), and VP6 (RNA-dependent ATPase and helicase) enclosed by layers of VP3 (subcore) and VP7 (core) (4, 6, 8, 9). The outer capsid is formed by two structural proteins, VP2 and VP5, that are responsible for cell attachment and entry (10–12). NS1, the largest of the nonstructural proteins, forms tubules in the cytoplasm of BTV-infected cells and is a positive regulator of viral protein synthesis (13–15). NS2 is a major component of viral inclusion bodies (VIB) which are the sites of BTV morphogenesis and RNA replication (16–18). NS3, the only BTV glycoprotein, is involved in virus exit from infected cells and is thought to play a role in counteracting the innate immune response of the host cell (19–21). The recently discovered NS4 is the smallest of the BTV proteins, and it also confers a replication advantage in cells in an antiviral state (5). At present, there are 26 different BTV serotypes circulating worldwide. The serotype is determined predominantly by VP2, which is the most variable of BTV proteins and a main target of neutralizing antibodies (22–25).

Bluetongue is remarkably variable in its clinical manifestations, which can range from asymptomatic infection to a lethal hemorrhagic fever (2, 26–28). This variability is due to several factors related both to the infected host and the virus (2, 29–33). Over the years, BTV has been used extensively as a model to study the orbivirus replication cycle, structural biology, and interaction with the host cell. However, we have little understanding of the molecular determinants of BTV virulence.

The northern European strain of BTV8 was the cause of one of the largest outbreaks in the history of bluetongue. The virus emerged in 2006 in an area between the Netherlands, Belgium, and northern Germany and then spread throughout the continent, causing high mortality in naive sheep flocks and, occasionally, also clinical disease in cattle (34–36). Interestingly, no clinical signs were detected in sentinel sheep when the virus reached northern Italy and Sardinia in 2008. Experimental infections with the northern European strain of BTV8 (BTV8_NET2006_) and the Italian strain of BTV8 (BTV8_IT2008_) confirmed a marked reduction in the virulence of the latter virus (33). A comparison of the consensus sequences of both viruses showed 24 nucleotide differences between the two strains, resulting in eight amino acid residue substitutions in VP1, VP2, VP4, VP6, NS1, and NS2.

Genetic drift occurring during natural transmission cycles plays a significant role in the diversification of BTV strains and their pathogenicity. Similarly, *in vitro* passage of BTV was shown to have an impact on virulence *in vivo* (37–39). In particular, strains isolated from severe clinical cases and consequently adapted to mammalian tissue culture have been reported to have reduced virulence in experimentally infected animals (33, 38). Interestingly, a decrease in pathogenicity was shown to occur even in minimally passaged strains and was attributed to a genetic bottleneck that occurs after a single passage in mammalian cells rather than to mutations in the consensus sequence (33). Conversely, live attenuated vaccines and tissue culture-adapted strains of BTV with a history of multiple passages *in vitro* show increased accumulation of nucleotide substitutions correlating with increasing numbers of passages in mammalian cells (39). Genomic segments 1, 2, and 8 (Seg1, Seg2, and Seg8, respectively, encoding VP1, VP2, and NS2) were shown to be consistently mutated in high-passage-number strains of BTV2, BTV4, and BTV9 viruses and to be attenuated in murine models of bluetongue (39).

Here, in order to determine the roles played by specific BTV genomic segments in virus adaptation to tissue culture and attenuation *in vivo*, we compared genetic and phenotypic differences between BTV8_NET2006_ minimally passaged in tissue culture, a derivative of this strain extensively passaged in cell culture, and 34 reassortants between the two viruses.

## MATERIALS AND METHODS

### Cell lines.

All cell cultures were grown at 37°C in 5% CO_2_ humidified atmosphere. BSR cells (a variant of BHK-21 cells; kindly provided by Karl-Claus Conzelmann) were propagated in Dulbecco's modified Eagle's medium (DMEM) supplemented with 10% fetal bovine serum (FBS) and 1% penicillin-streptomycin (P-S). Transfections in BSR cells were performed in DMEM with reduced FBS and no antibiotics. CPT-Tert cells (40) are sheep choroid plexus cells immortalized with simian virus 40 (SV40) T antigen and human telomerase reverse transcriptase. CPT-Tert cells were propagated in Iscove's modified Dulbecco's medium (IMDM) supplemented with 10% FBS and 1% P-S. The CHO cell line is derived from adult Chinese hamster ovary, and pgsA-745 (ATCC number CRL-2242) is a CHO-derived cell line deficient in xylotransferase that does not produce glycosaminoglycans (GAGs). Both cell lines were propagated in Ham's F-12 medium supplemented with 10% FBS and 1% P-S.

### Primary endothelial cell cultures.

Primary ovine aortic endothelial cells (OvEC) were isolated directly from aortas harvested from euthanized animals as recently described (41). Cells were maintained at 37°C in 5% CO_2_ and 3% O_2_ for a maximum of three passages.

### Virus strains.

BTV8_NET2006_ (Pirbright reference collection number NET2006/04) was originally isolated from a naturally infected sheep during the 2006 outbreak in northern Europe and has been previously described (5, 33). In this study, we refer to this virus as BTV8_L_. The subscript “L” is used to indicate that this virus has a low number of passages in cultures. BTV8_H_ (H, high passage number) was obtained by 65 serial passages of the BTV8_L_ strain in BSR cells, followed by plaque purification.

### Reverse genetics.

Plasmids used for the rescue of BTV8_L_ (resulting in rgBTV8_L_) were described previously (5, 42). Plasmids used for the rescue of BTV8_H_ by reverse genetics (resulting in rgBTV8_H_) were derived by site-directed mutagenesis of nonsynonymous sites in BTV8_L_. Reassortants between rgBTV8_L_ and rgBTV8_H_ are denoted with the name of virus forming the backbone (either BTV8_L_ or BTV8_H_) followed by the substituted segment marked with the “L” or “H” subscript to indicate the virus of origin. For example, BTV8_L_+S2_H_ is a reassortant containing the rgBTV8_L_ backbone with Seg2 from rgBTV8_H_. Rescued versions of both BTV8_L_ and BTV8_H_ (rgBTV8_L_ and rgBTV8_H_, respectively) and reassortants between both parental viruses were obtained using established procedures for BTV reverse genetics (5, 42, 43).

### Virus titrations and replication curves.

Titers of viral stocks were determined by standard plaque assays in CPT-Tert cells as previously described (25, 43). Virus replication in CPT-Tert cells and OvEC was assessed by infecting cells at a multiplicity of infection (MOI) of 0.01 and collecting supernatants at 2, 24, 48, and 72 h postinfection (p.i.). To compare replication kinetics of selected viruses in CHO and pgsA-745 cells, infections were performed at an MOI of 0.01, and supernatants were collected only at 72 h p.i. Supernatants were centrifuged for 5 min at 500 × *g* to remove cell debris and then titrated by endpoint dilution assays. Titers were expressed as the log_10_ 50% tissue culture infective doses/ml (TCID_50_/ml). All virus titration experiments were performed at least three times independently. Statistical calculations were carried out using GraphPad Prism.

### BTV genome sequencing.

The full genome sequence of BTV8_H_ was obtained using the Illumina platform. BSR cells were infected, and total RNA was extracted using TRIzol reagent (Invitrogen). Single-stranded RNA was precipitated using lithium chloride, and double-stranded RNA was harvested from the supernatant by precipitation with isopropanol in the presence of sodium acetate. Double-stranded RNA was used as a template for full-length amplification of cDNA (FLAC) by reverse transcription-PCR (RT-PCR) using established methods (44). Samples were analyzed using an Illumina Genome Analyser. The libraries were constructed from the PCR samples using a TruSeq DNA sample preparation kit (Illumina) according to the manufacturer's instructions. Briefly, the DNA samples were fragmented, the fragment was end repaired, and the 3′ ends were adenylated. After adaptor ligation steps, the fragments were purified by size selection on agarose gel, and the fragments containing adaptors on the 3′ and 5′ ends were enriched by PCR. Sequencing was performed on a GAIIX sequencer (Illumina) according to the manufacturer's protocol. Genomes were assembled using Maq software (45) with BTV8_L_ used as a reference sequence. The assemblies were manually curated using Tablet for sequence visualization (46), and consensus sequences were generated as Fasta files.

### *In vivo* experiments.

Animal experiments were carried out at the Istituto Zooprofilattico Sperimentale dell'Abruzzo e del Molise G. Caporale (Teramo, Italy) in accordance with locally and nationally approved protocols regulating animal experimental use (protocol no. 11427/2012). Experiments in sheep were conducted in an insect-proof isolation unit with veterinary care. Prior to the experiments the animals were confirmed to be seronegative for BTV using a blocking enzyme-linked immunosorbent assay (ELISA), as described previously (33). Groups (*n* = 5 animals per group) of Dorset Poll sheep were infected intradermally with a total of 2 × 10^6^ PFU (in 5 ml) of BTV8_L_ and BTV8_H_ by multiple inoculations in the inner leg and in the prescapular areas. Control animals were inoculated with 5 ml of mock-infected cell supernatant. Blood samples were collected from all animals daily for the first 15 days and at 17, 19, 21, and 28 days p.i. and then examined for the presence of viremia by quantitative RT-PCR (qRT-PCR). Sera were tested by virus neutralization assay for the presence of BTV-specific antibodies in the samples collected at 28 days p.i. All sheep were examined for the presence of clinical signs and fever, starting 1 week prior to inoculation and continuing for 15 days p.i., with further observations on days 17, 19, 21, and 28 p.i. Clinical signs were scored using a clinical reaction index (CRI) as previously described (33).

Transgenic mice deficient in type I interferon (IFN) receptor (129sv IFNAR^−/−^) were maintained at biosafety level 3. For each experiment, groups of adult mice matched for sex and age (*n* = 5 per group) were infected intraperitoneally or subcutaneously with 300 PFU or 3,000 PFU of virus or mock-infected cell culture medium as a control. Mice were examined for clinical signs daily until the experiment was concluded at 14 days p.i.

### Serum neutralization assays.

The presence of neutralizing antibodies was assessed by neutralization assays as previously described (33, 43).

### qRT-PCR.

Levels of viremia in infected sheep were assessed by qRT-PCR as described before (33). Red blood cells were lysed with water for 10 min on ice and centrifuged at 4°C for 10 min at 13,000 × *g*. Armored West Nile RNA (Asurage, USA) was spiked into each sample as an internal control for nucleic acid extraction. Total RNA was extracted using a High Pure nucleic acid extraction kit (Roche, Nutley, NJ) according to the manufacturer's instructions. For all samples, 250 ng of total RNA was amplified by one-step qRT-PCR using primers and probes for BTV Seg5. Armored RNA and β-actin were amplified as control reactions.

Levels of glyceraldehyde-3-phosphate dehydrogenase (GAPDH), β-actin, IFN-β, Mx1, and RSAD2 expression in infected OvEC were measured by qRT-PCR. Briefly, cells were seeded in 24-well plates and infected 48 h afterwards with rgBTV8_L_, rgBTV8_H_, or selected reassortants (MOI of 1). The medium was replaced after 1 h, and the cells were incubated for a further 17 h at 37°C. Next, supernatants were collected, and monolayers were directly lysed in 0.5 ml of TRIzol (Life Technologies). Phase separation was performed according to the manufacturer's instructions, whereupon the aqueous phase was mixed with ethanol and purified using an RNeasy minikit (Qiagen), including an RNase Free/DNase Set on-column DNase treatment step. Residual contaminating genomic DNA was removed using a Turbo DNA-Free kit (Ambion) according to the manufacturer's conditions. Reverse transcription was performed using 100 ng of RNA using random hexamers and SuperScript III (Life Technologies) for 1 h at 45°C. Quantitative PCR (qPCR) was performed using Brilliant III Ultra-Fast QPCR mastermix reagents (Agilent) and in-house designed primers/probes (sequences available upon request) targeting ovine GAPDH, β-actin, IFN-β, Mx1, and RSAD2. Samples were run on an Mx3005P PCR machine with rgBTV8_L_-infected cells set as a calibrator. GAPDH was used as normalizing gene against which fold induction was determined for IFN-β, Mx1, RSAD2, and β-actin.

### IFN protection assays.

Measurement of IFN levels in cell supernatants was based on methods described previously (5, 41). Briefly, OvEC were seeded in 24-well plates and infected after 2 days at an MOI of 1 with selected viruses. Medium was replaced after 1 h of incubation, and supernatants were collected 18 h after infection and treated for 20 min with UV light to inactivate infectious virus. CPT-Tert cells were seeded in 96-well plates, and 24 h later 2-fold serial dilutions of UV-treated supernatants or serial dilutions of a known concentration of universal interferon (UIFN) were added to the cells. After 24 h of incubation, supernatants were removed, and cells were infected with encephalomyocarditis virus (EMCV) and incubated for further 48 h. Cells were then inspected for EMCV-induced cytopathic effect. The levels of IFN in supernatants collected from BTV-infected OvEC were calculated based on the number of wells protected from EMCV-induced cell death compared to those of the UIFN control wells.

Cell protection by pretreatment with UIFN was performed using CPT-Tert cells. Cells were seeded in 24-well plates and 24 h later treated with 1,000 units of UIFN. After 18 h of incubation, UIFN was removed, and cells were infected with selected viruses at an MOI of 0.01. In parallel, untreated CPT-Tert cells were infected with the same set of viruses. Inocula were replaced by fresh tissue culture medium 1.5 h later. At 48 h p.i. supernatants were collected, and viral titers were determined by endpoint dilution assay and expressed as the log_10_ TCID_50_/ml.

## RESULTS

### Phenotype of tissue culture-adapted BTV8.

**FIG 1:**
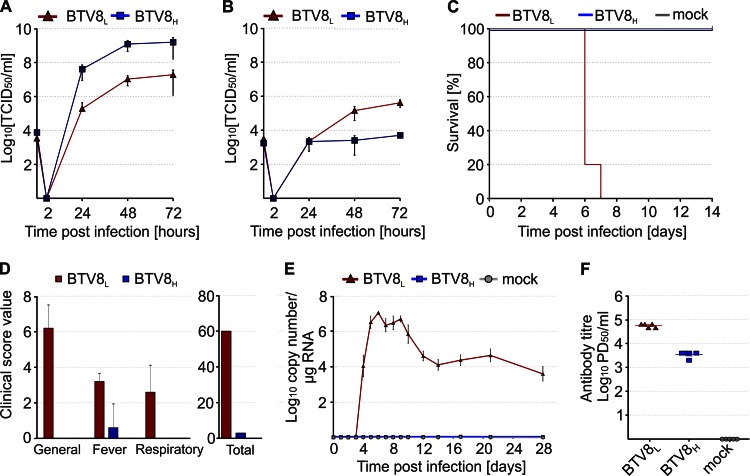
*In vivo* and *in vitro* phenotype of minimally passaged BTV8_L_ and tissue culture-adapted BTV8_H_. (A and B) BTV8_L_ and BTV8_H_ replication kinetics in ovine CPT-Tert cells (A) and primary ovine endothelial cells (OvEC) (B). Cells were infected with BTV8_L_ or BTV8_H_ at an MOI of 0.01. Supernatants were collected at 2, 24, 48, and 72 h p.i. and then titrated in BSR cells by limiting dilution analysis. Virus titers are expressed as log_10_(TCID_50_/ml). (C) Survival plot of IFNAR^−/−^ mice (*n* = 5 per group) infected with 300 PFU of BTV8_L_ and BTV8_H_ or mock infected. (D to F) Virulence of BTV8_L_ and BTV8_H_ in sheep. Clinical scores (D), viremia (E), and neutralizing antibodies (F) at 28 days p.i. were measured in infected and mock-infected sheep (*n* = 5 per group) as described in Materials and Methods. PD_50_, 50% protective dose.

BTV8_L_ was serially passaged 65 times in BSR cells and plaque purified. The resulting virus, BTV8_H_, and the parental BTV8_L_ were then titrated in sheep CPT-Tert cells and in primary ovine endothelial cells (OvEC). CPT-Tert cells are unable to initiate an IFN response following virus infection (5, 40), while OvEC are IFN competent (41). Both viruses replicated very efficiently in IFN-deficient CPT-Tert cells, but BTV8_H_ reached titers approximately 100-fold higher than its parental virus (Fig. 1A). Both viruses produced lower yields in OvEC than in CPT-Tert cells, and BTV8_L_ replicated better than BTV8_H_ in these cells (Fig. 1B).

Next, we determined the pathogenicity of BTV8_L_ and BTV8_H_ in IFNAR^−/−^ mice. BTV8_L_ was highly virulent in this mouse model, inducing 100% mortality within 7 days p.i., whereas BTV8_H_ was completely attenuated (Fig. 1C). Next, we assessed the virulence of BTV8_L_ and BTV8_H_ in sheep, the natural host of BTV infection, in order to characterize further the phenotype of these viruses *in vivo*. Sheep infected with BTV8_L_ showed fever and general clinical signs, including depression and mild respiratory distress. Viremia was detected in all BTV8_L_-infected animals (*n* = 5) from approximately day 4 p.i. until the end of the experiment at day 28 p.i. On the other hand, sheep infected with BTV8_H_ did not show any clinical signs of bluetongue or viremia (Fig. 1D and E). As expected, both BTV8_L_- and BTV8_H_-infected sheep developed neutralizing antibodies although the antibody titers were lower in the BTV8_H_-infected animals (Fig. 1F).

### Genotype and phenotype of rgBTV8_L_ and rgBTV8_H_.

**FIG 2:**
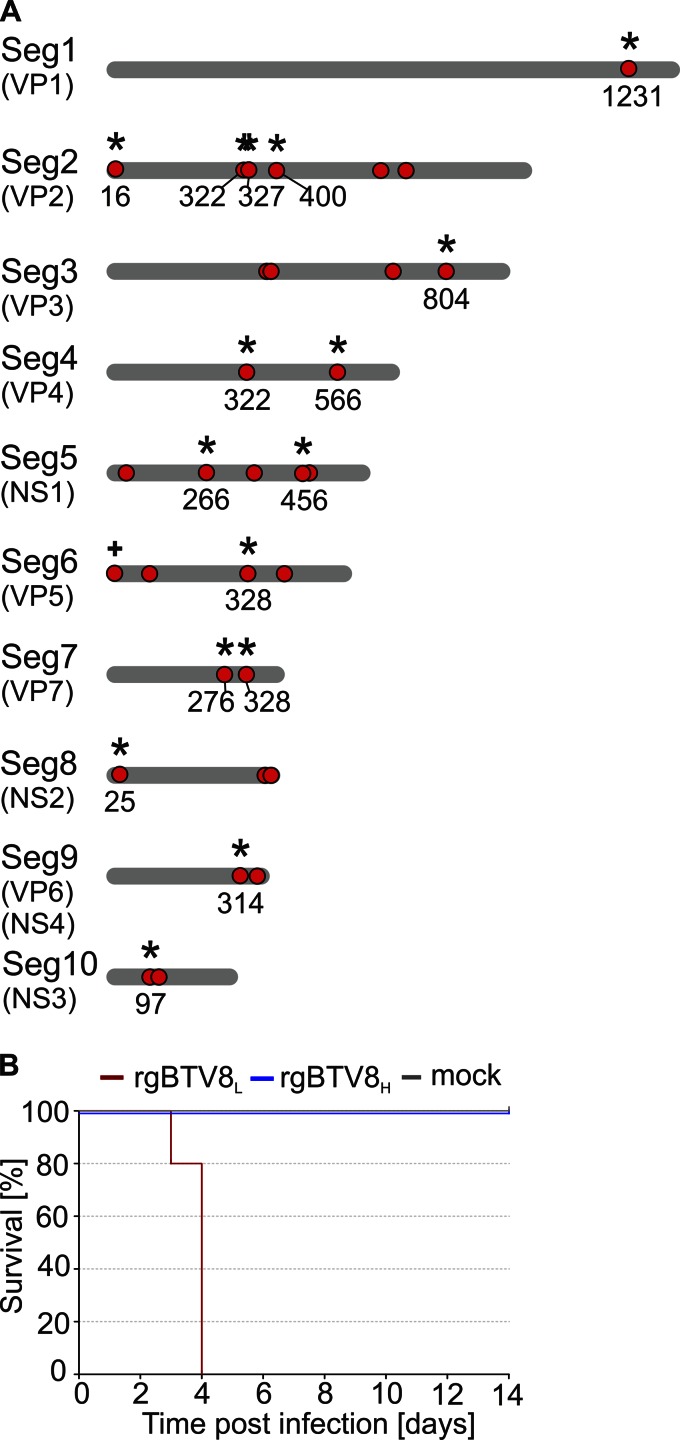
Genetic differences between BTV8_L_ and BTV8_H_ and virulence of rescued viruses in IFNAR^−/−^ mice. (A) Schematic representation of the 10 genomic segments of BTV8_L_ and BTV8_H_. Mutations in BTV8_H_ compared to the sequence of the minimally passaged BTV8_L_ are indicated with red dots. Nonsynonymous mutations are marked with asterisks, and the numbers relative to the mutated amino acid residues in the corresponding viral proteins are shown. The plus sign indicates a nucleotide insertion. The length of the schematic genome segments and the relative position of mutations are indicative only. (B) Survival plots of IFNAR^−/−^ mice (*n* = 5 per group) infected intraperitoneally with 300 PFU of rgBTV8_L_ or rgBTV8_H_.

The data obtained so far indicate that BTV8_H_ had accumulated mutations that affected virus replication in IFN-competent cells and resulted in attenuation *in vivo* both in IFNAR^−/−^ mice and in sheep. Full-genome sequencing of BTV8_H_ revealed 31 nucleotide mismatches compared to the sequence of BTV8_L_, out of which 16 resulted in amino acid residue substitutions (Fig. 2A). Nonsynonymous mutations were present in each of the 10 genomic segments and affected all characterized BTV proteins with the exception of NS4.

In order to identify BTV8 genomic segments affecting virulence, we rescued both BTV8_L_ and BTV8_H_ by reverse genetics. We reconstructed BTV8_H_ by substituting the 16 nucleotide mutations of BTV8_H_ into the BTV8_L_ vectors by site-directed mutagenesis. Hence, we rescued rgBTV8_L_ with the same nucleotide sequence as the original virus (BTV8_L_) while rgBTV8_H_ was identical to the original high-passage-number virus at the amino acid level but did not contain the silent mutations present in the genome of BTV8_H_. We then inoculated IFNAR^−/−^ mice with rgBTV8_L_ and rgBTV8_H_ in order to test whether the phenotypes of the rescued viruses were identical to those of the parental BTV8_L_ and BTV8_H_. Groups of five mice were inoculated intraperitoneally with 300 PFU of each virus and monitored over a 14-day period (Fig. 2B). All mice infected with rgBTV8_H_ survived the challenge while rgBTV8_L_ caused 100% mortality. These data showed that both rescued viruses retained the phenotypes of the original viruses.

### Pathogenicity of reassortants between rgBTV8_L_ and rgBTV8_H_.

**FIG 3:**
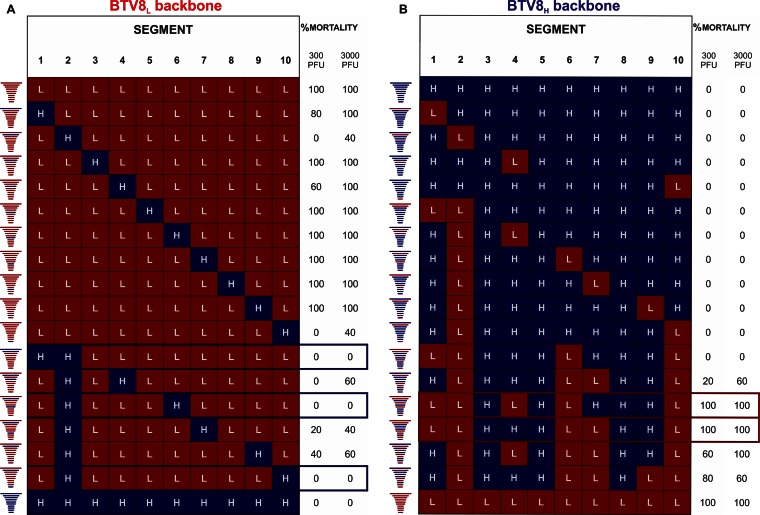
Virulence of BTV8_L_/BTV8_H_ reassortants in IFNAR^−/−^ mice. Multiple reassortant viruses were rescued using reverse genetics as described in Materials and Methods. Mortality of IFNAR^−/−^ mice (*n* = 5 per group) inoculated intraperitoneally with 300 or 3,000 PFU of individual reassortants within the BTV8_L_ or the BTV8_H_ backbone is shown. Note that the combination of segments that resulted in rescue of fully attenuated reassortants is shown in blue boxes. The combination of at least five segments of BTV8_L_ was required to recapitulate the fully virulent phenotype (shown in red boxes).

In order to define the genomic segments carrying attenuating mutations, we rescued in total 34 reassortants. A set of reassortants was based upon the BTV8_L_ backbone with a single genomic segment or a combination of segments from BTV8_H_. Four monoreassortants showed reduced mortality in IFNAR^−/−^ mice (Fig. 3A). In particular, BTV8_L_+S2_H_ and BTV8_L_+S10_H_ caused no mortality in mice at an inoculation dose of 300 PFU and caused 40% mortality with 3,000 PFU. Full attenuation using both inoculation doses was achieved with double reassortants BTV8_L_+S1/2_H_ (which contains Seg1 and Seg2 of BTV8_H_), BTV8_L_+S2/6_H_, and BTV8_L_+S2/10_H_. These data suggest that VP2 and NS3 primarily, in addition to VP5 and VP1, were major factors of BTV8_H_ attenuation in this mouse model. We next aimed to identify the minimal number of BTV8_L_ segments that would restore full virulence to a reassortant based upon the BTV8_H_ backbone (Fig. 3B). None of the single or double reassortants was virulent in IFNAR^−/−^ mice. Furthermore, BTV8_H_+S1/2/6/10_L_ also possessed an attenuated phenotype. These data suggested that other viral proteins besides VP1, VP2, VP5, and NS3 play a role in the virulence of BTV8_L_. To achieve 100% mouse mortality at an inoculation dose of either 300 or 3,000 PFU, it was required that at least five proteins of BTV8_H_ be replaced by the BTV8_L_ equivalents, including either VP4 or VP7 in conjunction with the aforementioned VP1, VP2, VP5, and NS3 (Fig. 3B).

### Replication kinetics of BTV8_L_ monoreassortants in CPT-Tert cells.

**FIG 4:**
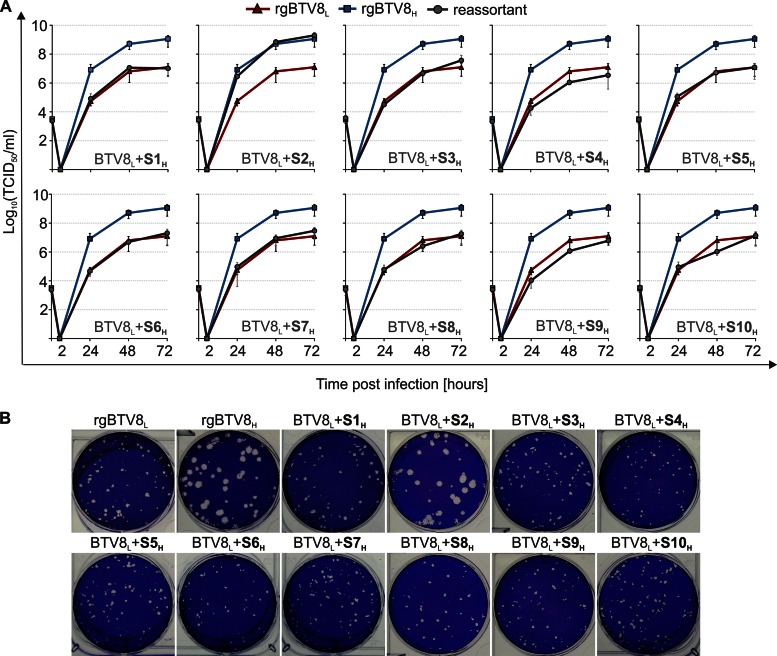
*In vitro* replication properties of BTV8_L_/BTV8_H_ monoreassortants. (A) Growth curves of parental rgBTV8_L_, rgBTV8_H_, and monoreassortants containing the BTV8_L_ backbone (gray circle) in CPT-Tert cells. Monolayers were infected with the indicated viruses at an MOI of 0.01, and supernatants were collected at 2, 24, 48, and 72 h p.i. Viral titers were determined by endpoint dilutions. All reassortants, with exception of BTV8_L_+S2_H_, showed replication kinetics similar to those of the parental rgBTV8_L_. (B) Plaques produced in CPT-Tert cells by parental rgBTV8_L_ and rgBTV8_H_ and derived monoreassortants at 48 h p.i. Note the increased plaque size in rgBTV8_H_ and BTV8_L_+S2_H_.

As shown above, the replacement of specific genome segments of BTV8_L_ with the homologous segments from BTV8_H_ resulted in attenuation *in vivo*. To establish whether the replication kinetics of these reassortants were compromised *in vitro*, we assessed the full set of 10 monoreassortants containing the BTV8_L_ backbone in CPT-Tert cells (Fig. 4A). Overall, 9 of the 10 monoreassortants displayed similar replication kinetics to those of the parental rgBTV8_L_. Remarkably, BTV8_L_+S2_H_ showed replication kinetics essentially identical to those of rgBTV8_H_ and reached over 100-fold higher titers than rgBTV8_L_ at 72 h p.i. Both rgBTV8_H_ and BTV8_L_+S2_H_ produced larger plaques than those induced by rgBTV8_L_ and all the other monoreassortants (Fig. 4B). Overall, these data indicated that VP2 was the sole determinant of increased replication efficiency of BTV8_H_ *in vitro* in CPT-Tert cells.

**FIG 5:**
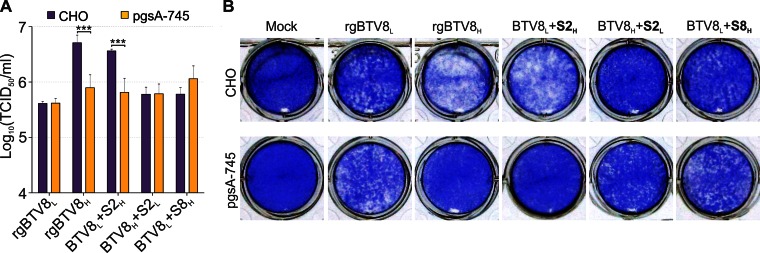
Segment 2 of rgBTV8_H_ favors replication in cells expressing glycosaminoglycans. (A) Viral titers produced in CHO and pgsA-745 cells infected with rgBTV8_L_, rgBTV8_H_, and reassortant viruses (MOI of 0.01). Supernatants were collected at 72 h p.i. and titrated in BSR cells by limiting dilution analysis. Mean values from three experiments performed in duplicate are shown (error bars correspond to standard deviations). Note that significant differences were observed between titers produced in CHO and pgsA-745 cells by BTV8_L_+S2_H_ and BTV8_H_+S2_L_ (***, *P* < 0.001; two-way analysis of variance followed by a Bonferroni test to identify interactions). (B) Cytopathic effect in CHO and pgsA-745 cells infected with rgBTV8_L_, rgBTV8_H_, and reassortant viruses (MOI of 0.01). Cells were stained with crystal violet at 72 h p.i.

Cells cultured *in vitro* tend to display increased expression of glycosaminoglycans (GAGs) at the cell membrane. Interestingly some viruses, like foot and mouth disease virus, (47) show an increased affinity for heparan sulfate after being passaged *in vitro*. We hypothesized that VP2 might have acquired higher affinity for GAGs during passage in tissue culture, given that VP2 is the main determinant of BTV serotype and mediates viral entry (22, 25, 48). Therefore, we performed viral replication kinetic assays in CHO cells and in a derived cell line (pgsA-745) deficient in xylotransferase and thus lacking heparan sulfate GAGs. rgBTV8_L_ and a BTV8_H_ reassortant with the VP2 of BTV8_L_ (BTV8_H_+S2_L_) grew equally well in both cell lines. However, rgBTV8_H_ and BTV8_L_+S2_H_ reached approximately 10-fold higher titers in CHO cells (*P* < 0.001) (Fig. 5A) and induced a more pronounced cytopathic effect (Fig. 5B) than rgBTV8_L_ and BTV8_H_+S2_L_, respectively. Hence, BTV8_H_ VP2 has a higher affinity for GAGs and facilitates BTV8 replication *in vitro* but not *in vivo*.

### Replication of rgBTV8_L_, rgBTV8_H_, and BTV8 monoreassortants in IFN-competent OvEC.

**FIG 6:**
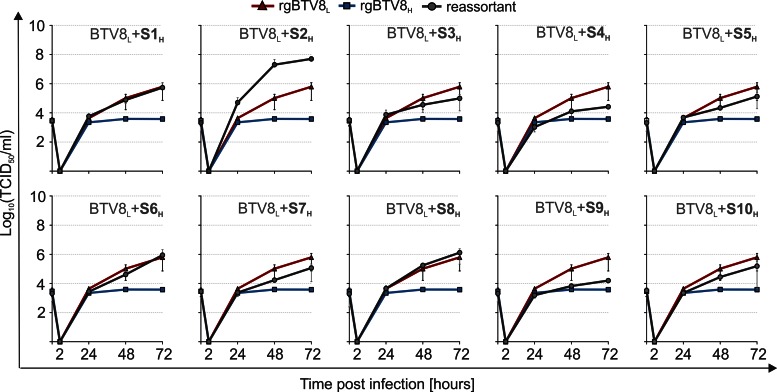
Replication kinetics of parental and monoreassortant viruses in primary OvEC. OvEC were infected at an MOI of 0.01 with rgBTV8_L_, rgBTV8_H_, and monoreassortants containing the BTV8_L_ backbone (gray circle). Viral titers were determined at the specified time points by limiting dilution assays. Each panel shows growth curves of both parental viruses and a specific monoreassortant (as labeled).

BTV8_H_ does not replicate efficiently in IFN-competent primary OvEC. Thus, we further explored this model in order to characterize the interplay between rgBTV8_L_/rgBTV8_H_ genomic segments and the cell-autonomous innate immune system. First, we compared the replication kinetics of BTV8_L_ and the BTV8_H_ monoreassortants in OvEC (Fig. 6). BTV8_L_+S2_H_ reached higher titers in culture than the other viruses tested, which suggested that mutations in VP2_H_ conferred an advantage to the replication of this reassortant *in vitro*, irrespective of the ability of the cell to produce IFN. Several reassortants demonstrated delayed growth compared to that of rgBTV8_L_. In particular, BTV8_L_+S4_H_ and BTV8_L_+S9_H_ yielded substantially lower titers than the parental BTV8_L_ at 48 and 72 h p.i. (Fig. 6). None of the monoreassortants, however, replicated as poorly as rgBTV8_H_, which indicated that the growth restriction of this virus was the cumulative result of mutations in several of its genome segments.

**FIG 7:**
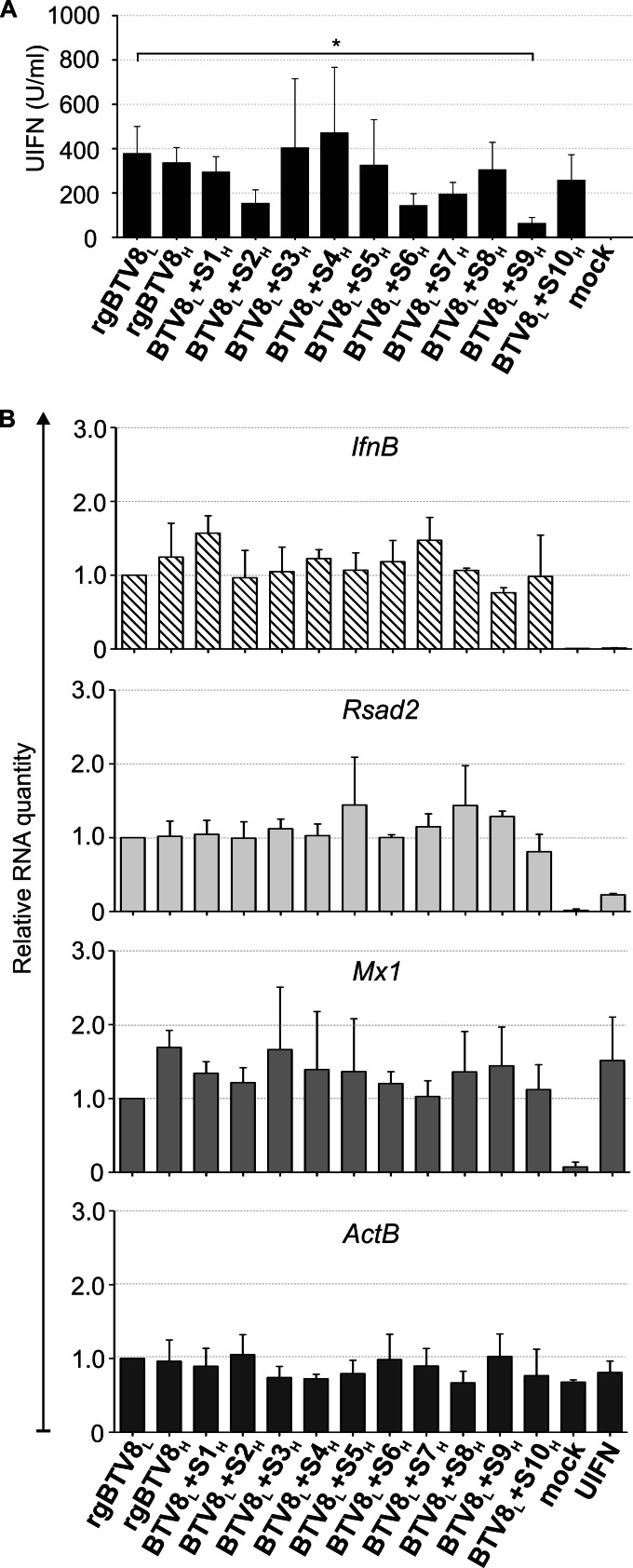
IFN production and gene expression induced by infection of OvEC by rgBTV8_L_, rgBTV8_H_, and BTV8_L_/BTV8_H_ monoreassortants. OvEC were infected with rgBTV8_L_, rgBTV8_H_, and monoreassortants within the BTV8_L_ backbone (MOI of 1). (A) IFN protection assay. Supernatants were collected at 18 h p.i., inactivated by UV treatment, and used in a biological assay to estimate the amount of IFN present, as described in Materials and Methods. The only major differences were observed in cells infected with BTV8_L_+S9_H_, where the amount of IFN released was significantly lower than what was found in cells infected with rgBTV8_L_ (*P* < 0.05; one-way analysis of variance followed by Dunnett's multiple-comparison test to dissect individual interactions). (B) IFN-β, ActB, RSAD2, and Mx1 gene expression. mRNA was measured by qPCR in OvEC at 18 h p.i. with parental and reassortant viruses (MOI of 1) as described in Materials and Methods. Mock-treated and UIFN-treated cells were used as controls. Panels show gene expression relative to rgBTV8_L_ and normalized to GAPDH gene levels.

Next, we wanted to establish whether BTV8_H_ was a more potent IFN inducer than the parental BTV8_L_. To this end, we measured IFN production in the supernatants of OvEC infected with the same set of viruses as above. rgBTV8_H_ and most of the monoreassortants induced similar amounts of IFN. However, statistically significant differences (*P* < 0.05) were observed between rgBTV8_L_ and BTV8_L_+S9_H_ (Fig. 7A).

We also assessed the relative quantities of the IFN-β mRNA, selected IFN-stimulated gene (ISG) mRNAs (Mx1 and RSAD2), and β-actin in OvEC infected with either rgBTV8_H_, rgBTV8_L_, or one of the various monoreassortants as above. Mock-infected cells and cells treated with universal IFN (UIFN) were used as controls. We detected no IFN-β mRNA in either of the control samples, while it was readily detectable at similar levels in cells infected with rgBTV8_L_, rgBTV8_H_, and the rgBTV8_L_/rgBTV8_H_ monoreassortants (Fig. 7B). Mx1 and RSAD2 were also upregulated in virus-infected cells, but no significant differences were found between cells infected with the various monoreassortants and the parental viruses (Fig. 7B). β-Actin levels were consistently uniform in all samples analyzed.

### Effect of IFN pretreatment on the replication of rgBTV8_L_, rgBTV8_H_, and rgBTV8_L_/rgBTV8_H_ monoreassortants.

**FIG 8:**
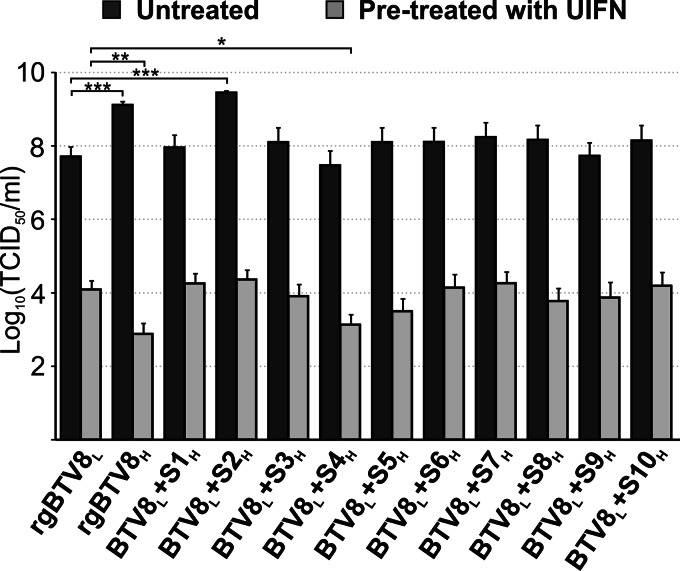
Replication of rgBTV8_L_, rgBTV8_H_, and BTV8_L_/BTV8_H_ monoreassortants in CPT-Tert cells pretreated with universal IFN (UIFN). Viral titers produced in untreated and IFN-pretreated CPT-Tert cells by parental and reassortant viruses at 48 h p.i. at an MOI of 0.01. Mean values from three experiments performed in duplicate are shown (error bars correspond to standard deviations). *, *P* < 0.05; **, *P* < 0.01; ***, *P* < 0.001 (one-way analysis of variance followed by Dunnett's multiple-comparison test to dissect individual interactions).

The data described above, showed that the reduced replication kinetics of rgBTV8_H_, BTV8_L_+S4_H_, and BTV8_L_+S9_H_ in OvEC were not due to increased IFN induction in these cells. In light of these data, we wanted to establish whether rgBTV8_L_, rgBTV8_H_, and the rgBTV8_L_/rgBTV8_H_ reassortants were better or worse equipped to overcome restriction in cells already in an antiviral state prior to infection. For these experiments, we used CPT-Tert cells as they do not produce IFN but can respond to IFN treatment (5, 40). We pretreated CPT-Tert with either 1,000 units of UIFN or control medium for 18 h prior to infection with rgBTV8_L_, rgBTV8_H_, and monoreassortants with a BTV8_L_ backbone at an MOI of 0.01. At 48 h p.i. supernatants of infected cells were collected and titrated by endpoint dilution analysis. Comparison of viral yields in cells treated and untreated with UIFN showed that the replication of all viruses was inhibited by at least 100-fold by UIFN (*P* < 0.001) (Fig. 8). The reduction in yield of rgBTV8_L_ in UIFN-treated cells compared to that in untreated cells was approximately (5 × 10^3^)-fold. Strikingly, this ratio was more than a millionfold (1.7 × 10^6^) for rgBTV8_H_. In UIFN-treated cells, most reassortants reached yields similar to those obtained by rgBTV8_L_ under the same conditions. A notable exception was BTV8_L_+S4_H_, which had yields 10-fold lower than those of rgBTV8_L_ (*P* < 0.05) in the UIFN-treated samples. BTV8_L_+S2_H_ showed the highest degree of inhibition in treated CPT-Tert cells among all 10 monoreassortants. However, the yield of BTV8_L_+S2_H_ in treated cells was equivalent to the one obtained by rgBTV8_L_ under the same conditions.

## DISCUSSION

Several BTV incursions have occurred in Europe in the last 20 years. Some strains, such as the northern European BTV8 strain used in this study, have caused major animal health and economic problems while others did not cause any clinical signs in the field (49, 50).

Although the concept of BTV serotype/strain-related virulence is often quoted in the literature, only a few studies have attempted to understand what the viral molecular determinants that underscore this property are (29, 39, 51). In this study, we aimed to map the molecular determinants of virulence of the northern European strain of BTV8. We found that nonsynonymous mutations in four segments (Seg1, -2, -6, and -10) encoding VP1, VP2, VP5, and NS3 contributed to the attenuation of BTV8 *in vivo* in IFNAR^−/−^ mice. Reassortants where segments encoding the VP2 or NS3 of the attenuated rgBTV8_H_ were used in the context of the virulent rgBTV8_L_ backbone caused no mortality in mice inoculated at the lower dose (300 PFU) used in this study. The combination of Seg2 with either Seg1, Seg6, or Seg10 of rgBTV8_H_ in the context of the rgBTV8_L_ backbone resulted in a fully attenuated reassortant that was nonpathogenic at either the low or high doses.

The multigenic nature of BTV attenuation has been previously suggested by us as we showed that tissue culture-attenuated strains of different BTV serotypes that show reduced virulence in IFNAR^−/−^ mice tend to accumulate mutations consistently in segments 1, 2, and 8 (39). Interestingly, Seg2 with either Seg1, Seg6, or Seg10 of rgBTV8_L_ did not restore virulence in the rgBTV8_H_ backbone. The fact that a degree of virulence in rgBTV8_H_ was achieved only after combining Seg7 with Seg2, Seg6, and Seg10 of rgBTV8_L_ might suggest that interactions between specific viral proteins (or genomic segments) could play a role in the pathogenicity of BTV. Both VP2 and VP5 have previously been shown to interact with VP7 trimers in BTV virions (52). Additionally, other studies demonstrated the importance of NS3 interactions with outer capsid proteins in virus trafficking and assembly (19, 53). It is therefore possible that while mutations in high-passage-number VP5 or VP7 did not directly affect the functions of these proteins, they did influence virus virulence through their interactions with VP2 and NS3.

Interestingly, two mutations found in VP2 of BTV8_H_ (positions 321 and 328) were located in the same region that was previously identified as being associated with attenuated BTV strains and as a target for neutralizing antibodies (39, 54, 55). This external and highly exposed area of VP2 was also implicated in attachment to a host cell receptor, and the mutations that arise in this region could be due to the changes in affinity for binding to specific ligands (10). Cryo-electron microscopy showed that VP2 possesses a sialic acid binding region located in its hub domain, which is one of two sites suggested to interact with the cell surface receptor (10). An increased affinity for GAGs has often been cited in the context of tissue culture-adapted strains (47, 56–58). Here, we found that BTV8_H_ or reassortants with the VP2 of BTV8_H_ had, indeed, a greater affinity for GAGs and were attenuated in IFNAR^−/−^ mice. Previous studies have shown that viruses acquiring mutations conferring a higher affinity for heparan sulfate proteoglycans are often attenuated *in vivo* (58–62).

BTV is a potent inducer of type 1 IFN *in vivo* and *in vitro* (5, 21, 63–66). IFNAR^−/−^ mice, due to the lack of expression of the type 1 IFN receptor, are a suitable tool to study determinants of viral virulence unrelated to IFN expression (39, 67, 68). However, it is most likely that other viral factors that are used to counteract the IFN system contribute to BTV virulence. Pathogenicity studies of multiple reassortants in sheep are not feasible; hence, we used primary OvEC as an *in vitro* surrogate model to identify the genome segments that confer higher sensitivity to the cell-autonomous IFN response. BTV8_H_ (and its derivative rescued by reverse genetics) replicated poorly in primary cells. These data showed that the IFN response played a role in the inhibition of BTV8_H_ replication. Both BTV8_L_ and BTV8_H_ induced similar amounts of IFN in infected primary cells. These data suggest that primary OvEC sense and respond to infection with both viruses in a similar manner but that BTV8_L_ was able to counteract this response better than BTV8_H_.

Most of the BTV8_L_ monoreassortants containing BTV8_H_ genomic segments were able to replicate to similar titers as the parental low-passage-number virus in primary cells. Two notable exceptions included BTV8_L_+S4_H_ and BTV8_L_+S9_H_. However, the VP4 (encoded by Seg4) of BTV8_H_ also influenced the viral phenotype in IFNAR^−/−^ mice, suggesting that mutations in this protein could also have affected its functions unrelated to the type I IFN response. VP4 of BTV acts as a capping enzyme, and therefore the mutations in VP4_H_ and, in particular, the mutation in the amino acid residue at position 332 of the predicted 2′-*O*-methyltransferase (2′ OMTase) domain could have an adverse effect on the efficiency of viral mRNA capping (69, 70). Moreover, recent studies have shown that viral mRNA that lacks 2′-O-methylation at its 5′ cap structure induces a more potent innate immune response through MDA5 activation or direct interactions with proteins members of the IFIT (interferon-inducible protein with tetratricopeptide repeats) family (71–73). An inefficient capping mechanism would therefore explain the slight, yet consistent, decrease in virus yields reached by BTV8_L_+S4_H_ in IFN-deficient CPT-Tert cells and the more pronounced growth inhibition in OvEC. This hypothesis was further supported by comparing the growth kinetics of monoreassortants in the BTV8_L_ backbone in CPT-Tert cells either untreated or pretreated with UIFN. Of all reassortants, BTV8_L_+S4_H_ showed the lowest yields in UIFN-pretreated cells, which suggested that VP4_H_ was one of the proteins contributing to the inability of rgBTV8_H_ to replicate in cells primed with IFN. These data are in concordance with other studies showing that West Nile virus, coronaviruses, and poxviruses mutants with deficient 2′ OMTase activity were not able to escape IFIT-2 induced restriction in cells stably expressing IFIT-2 (72). It is therefore possible that through viral mRNA capping, the VP4 of BTV8 could play a role in evading host restriction factors in order to allow the virus to replicate in host cells in an antiviral state.

BTV8_L_+S9_H_ also replicated less efficiently than the parental virus in primary endothelial cells. VP6 is encoded by Seg9 of the BTV genome, as is NS4, which is a nonstructural protein shown to counteract the IFN system (5; unpublished data). We found no mutations in the NS4 open reading frame, but we detected a nonsynonymous mutation in VP6 (RNA-dependent ATPase and helicase). We are not aware of viral helicases being involved in interactions with the innate immune system. We noticed a slight reduction in the replication kinetics of BTV8_L_+S9_H_ (compared to those of BTV8_L_) in CPT-Tert cells. Therefore, this reassortant might possess a suboptimal replication capacity that is amplified in the IFN-competent OvEC.

It is important to stress that although the BTV monoreassortants displayed an array of intermediate growth patterns in OvEC, none of them replicated as poorly as rgBTV8_H_. This indicated that mutations in segments other than Seg4 and Seg6 contributed to the restricted replication of the high-passage-number virus. Altogether, these data reinforce the concept that passaging viruses in an environment with no constraints from the IFN system allows greater flexibility of the viral genome, which in turn leads to the emergence of viruses with optimal replication efficiencies. The mutations that arise under such conditions might not necessarily involve major IFN antagonists but can involve proteins that are normally fine-tuned to evoke a minimal immune response while allowing sufficient (yet suboptimal) transmission in the natural host (74). A recent study looking into interserotype determinants of BTV pathogenesis in sheep showed that replacement of BTV1 Seg2, -6, and -10 with homologous BTV8 segments resulted in reassortants that showed a similar phenotype in sheep as BTV8 (51). However, this study was performed using BTV1 and BTV8 serotypes that are both virulent in sheep. Consequently, a clear distinction between levels of pathogenicity of the parental viruses and their reassortants was difficult to assess.

In conclusion, our data show that multiple genome segments determine the virulence of BTV8. Our study reinforces the concept that a constellation of genome segments determines the virulence of viruses with a segmented genome, as suggested in other studies in rotaviruses, influenza viruses, and bunyaviruses (74–82).

VP2 and NS3 were found to be primary determinants of BTV8 pathogenesis although VP1, VP5, VP4, VP6, and VP7 were also found to contribute to virulence. Given the high diversity of BTV, it is possible that different determinants of pathogenicity will be found in other serotypes. More studies aimed to map the molecular determinants of BTV virulence, using different strains/serotypes and experimental systems, will help to build an intellectual framework to characterize thoroughly BTV pathogenesis. The possibility to determine the pathogenicity of viral isolates on the bases of their genome sequences could help in designing control strategies that fit the risk posed by new emerging viruses.
